# In Situ Free Radical Growth Mechanism of Platinum Nanoparticles by Microwave Irradiation and Electrocatalytic Properties

**DOI:** 10.1186/s11671-016-1653-9

**Published:** 2016-10-14

**Authors:** Gajendra Kumar Inwati, Yashvant Rao, Man Singh

**Affiliations:** 1 Centre for Nanosciences, Central University of Gujarat, Gandhinagar, 382030 India; 2 School of Chemical Sciences, Central University of Gujarat, Gandhinagar, 382030 India

**Keywords:** Nanostructures, Chemical synthesis, Electron spin resonance, Catalytic properties

## Abstract

Microwave irradiation was employed for spherical-shaped platinum nanoparticle (Pt NPs) preparation. Spherical Pt NPs indexed with (111) facets were prepared using Pt(II) precursor salt, glycerol as solvent and reducing agent, and polyvinylpyrrolidone (PVP) as a shape directer under microwave irradiation for 3–5 min at 300 °C. Electron spin resonance (ESR) peak at 336.000 mT (milli Tesla) confirmed the free radical formation from aqueous glycerol solution which acted as reducing species under microwave. The 2–8-nm diameter of particles was obtained by high-resolution transmission electron microscope. Dynamic light scattering was used to optimize the microwave dose followed by 33 and 48 nm size and 51 and 67 mV zeta potential of Pt NPs, respectively. The PVP was demonstrated as shape controlling agent investigated by Fourier transmission infrared spectroscopy (FTIR). The electrocatalytic performance of as-prepared Pt colloids was investigated using cyclic voltammetry which showed a higher catalytic activity for ethanol redox reaction.

**Electronic supplementary material:**

The online version of this article (doi:10.1186/s11671-016-1653-9) contains supplementary material, which is available to authorized users.

## Background

Metallic nanostructures are widely applicable in various applications due to their dimensional confinement and active surface sites at nanoscale. The structurally engineered and functionalized nanomaterials have extremely unusual properties than bulk. Thereby, metallic, multimetallic, and metal oxide-based nanoscopic structures have gained much attention in use of chemical, physical, biological, and material science [1–7]. In novel metals, platinum is one of the exceptional metals due to its potential applications in electrocatalysis, organic synthesis, photovoltaic, and fuel cells [8–10]. To structured uniformed catalytic captivities with controlled shape and size at nanoscale, researchers have developed several methods to synthesize monodisperse platinum nanoparticles. Platinum is used as the most promising candidate in energy storage and conversion devices, but the cost of Pt is extremely high due to its rarity and higher demands as wider and safer nanocatalysts [11]. Therefore, developing of enhanced metallic electrocatalysts using less toxic reagents with simplest and efficient method is still a biggest challenge.

Presently, chemical reduction is one of the efficient processes to synthesize the Pt NPs with tuneable morphology [12–14]. Till date, several methods have been developed and reported for Pt NPs production with modified surface structures [15–17]. However, two facile and eco-friendly approaches were mentioned to generate Pt NPs. The one is traditional solvothermal, and the second is a microwave-assisted method. Hence, microwave-assisted synthesis is a faster heating chemical way than conventional heating to elongate chemical reactions extensively useful for nanomaterial preparation [18–20]. Thus, the short-time microwave irradiation, faster reaction rate, and fine distribution of NPs are the superiorities against conventional heating process [21, 22]. Furthermore, Grace et al. [23] prepared uniform and stable polymer-protected Pt, Pd, Ag, and Ru nanoparticles using microwave heating with polyol as solvent and reducing agents, but they are not given any mechanism related to free radical formation from polyol under microwave irradiation. Further, Komarneni [24] and Yanagida [25] have developed microwave-assisted method which is a longer time consuming and comparably complicated for Pt and Ag formation. However, they have not reported the free radical generation from the used solvent contrary to our approach which have successfully developed the Pt NPs. Additionally, EI-Sayed et al. [26] have reported the reduction of Pt electrocatalysts as polydisperse Pt NPs with altered morphology in a hydrogen-reducing way, using sodium polyacrylate as a capping agent. It was also comparably long time process.

Hence, it would be useful to achieve the Pt NPs by a systematic and mechanistic route such as in aqueous glycerol under microwave heating followed by free radical formation. The electrocatalytic activity for ethanol also investigated and inferred a prepared Pt colloidal liquid. Thereby, the glycerol is considered as green and economic reducing agent and solvent to generate rapid Pt NPs via free radical generation. This method needs hardly 3 to 5 min for complete synthesis without using any complication. Therefore, our approach could be an advance research over the existed redundant ways as Pt NPs.

### Experimental Section

Potassium tetrachloroplatinate(II) (K_2_PtCl_4_, 99.9 %, Pt 46.9977 %) was taken as precursor salt, and glycerol (99.9 % Sigma Aldrich) was chosen as both solvent and reducing agent. Poly(*N*-vinylpyrrolidone) (PVP; Avg. Mw = 40,000) was selected as capping agent in our experiment for synthesis of Pt NPs by microwave-assisted synthesis. The wt.% PVP of average Mw was first dissolved in water, and the glycerol was added drop wise in it followed by sonication for 10 min. A 1 M of K_2_PtCl_4_ was added in aqueous glycerol with continuous sonication for 5 min. For the Pt(II) reduction, the as prepared mixture was introduced into a microwave reactor (Anton Paar, Synthos 3000) for irradiation at 280 W in a pulse mode with pulse duration of 20 s (ON 15 s, OFF 5 s). Pressure and temperature profiles were recorded in reactor during these two modes of irradiation (Additional file 1: Figure S1). The detailed conditions associated with preparation are summarized in Additional file 1: Table S1. After completing the reaction, an irradiated mixture was cooled at room temperature and then the Pt colloids were taken for characterizations and electrocatalytic study. The new and novel reduction mechanism for preparing the Pt NPs using glycerol under microwave irradiation was developed. This method has successfully developed the free radicals which were confirmed by ESR study (Fig. 5). The free radicals have effectively reduced the platinum cations to platinum NPs which were compared with the metallic nanocomposite [27].

### Characterization Techniques

High-resolution transmission electron microscopy (HRTEM) measurements were taken by JEOL JEM 2100F microscope operating at 200 kV. The samples were prepared by placing a drop of Pt nanoparticles dispersed in ethanol onto a carbon-coated copper grid and dried for whole night in RT (25 °C). EDX spectroscopy was used to investigate elemental compositions, and the EDX was equipped with HRTEM. FTIR spectra were recorded on a (FTIR, Perkin Elmer) spectra scanned from 400–4000 cm^−1^ in FTIR range, spectrometer in the transmission mode with a spectral resolution of 4 cm^−1^ and 32 scans. Specimens for infrared measurements were prepared by mixing several drops of ethanol/water mixed solution containing Pt NPs with KBr plate followed by baking with infrared light. UV-vis absorption spectra of samples were measured on a (spectro 2060 plus spectrophotometer over 200–800 nm using 1 cm path length cuvette-UV analysis) equipped with a 1-cm path length quartz cuvette. The particle size distribution and zeta potential were measured with a dynamic light scattering (DLS) (Microtrac Zetatrac, U2771, DLS XE-70, Park System equipment) at RT. The reaction solution used for measured size distribution hence the liquid viscosity was set to 1.5 cp and the liquid index of refraction was 1.359. Electron spin resonance (ESR) spectra of free radical formation during the irradiation were collected at liquid N_2_ environment. The samples were placed in a 4.5-mm diameter quartz Dewar tube. The spectra of continuous-wave ESR (CW-EPR) at 9439.939 MHz, MOD 100.00 KHz, microwave power 3 mW, and the sweep time was 2 min.

## Results and Discussion

### UV-Vis Spectra Analysis

**Fig. 1:**
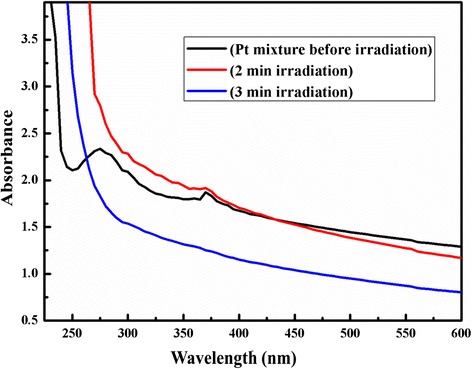
UV-vis spectra at before and after irradiation for 2 and 3 min

Figure 1 shows UV-vis spectra of K_2_PtCl_4_ in aqueous glycerol before and after microwave irradiations. It could be seen that the UV-vis spectra exhibit an absorption band around 275 nm with a weak at 370 nm before irradiation. This absorption band is attributed due to a charge transfer from Cl^−^ ligand to Pt^2+^ ions [28]. Also, the absorption band disappeared after irradiation at 280 W for short time duration (3 min). This band disappeared may be due to the complete reduction of platinum metal ions into neutral Pt(0). Thus, there was no further charge transfer possible from ligand to metal ions [29]. Consequentially, by UV-vis analysis, it is confirmed that the Pt(II) was converted into Pt(0) under applied microwave irradiation at 3 min.

### FTIR Spectra Analysis

**Fig. 2:**
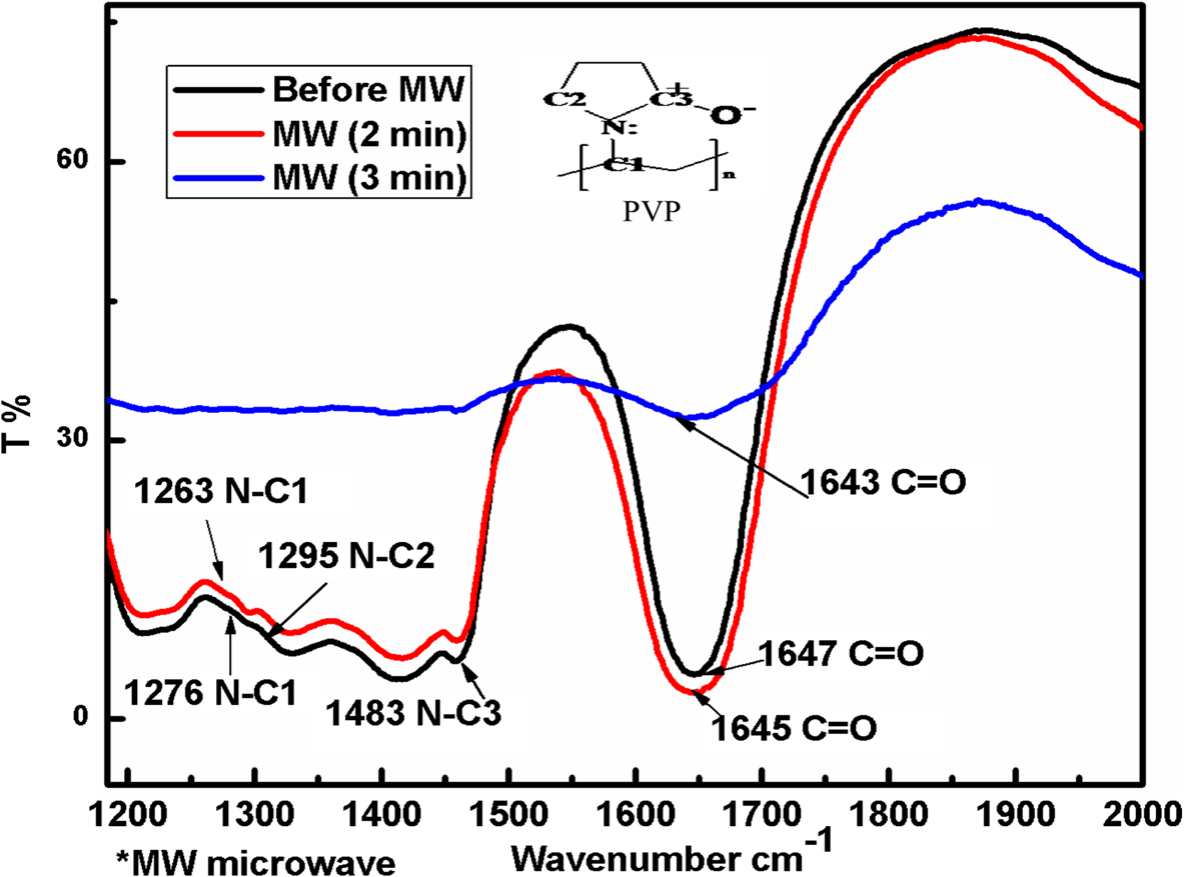
FTIR spectra of prepared mixture before and after irradiation at various times

Figure 2 shows FTIR spectra before and after microwave irradiation of Pt(II) reaction solution. The Pt(II) aqueous glycerol with PVP showed the similar FTIR spectra as pure PVP at initial time means there is no significant change in C=O and N–C stretching as is shown in Fig. 2. After 3-min irradiation, the C=O vibrational band showed a red shift from 1647, 1645, and 1643 cm^−1^ frequencies due to chemisorption of soft part of O atom of PVP with Pt solid surface [30, 31]. Further, it is found that the N–C (292 kJ/mol) have less bond energy than C–C (348 kJ/mol), C=O (351 kJ/mol) and C–H (391 kJ/mol) bonds [32]. Here, the N–C_1,2,3_ vibration bands are found at 1276, 1295, and 1483 cm^−1^ before irradiation but after 3-min irradiation, these vibrational frequencies were not appeared may be due to dissociation of N–C bond of N–C=O and N–C–C in PVP structure. So, it is concluded that the PVP adsorbed with both O atoms and N atoms at 3-min microwave irradiation which protect the Pt NPs surface growth.

### Optimization of MW Reactions Using DLS

**Fig. 3:**
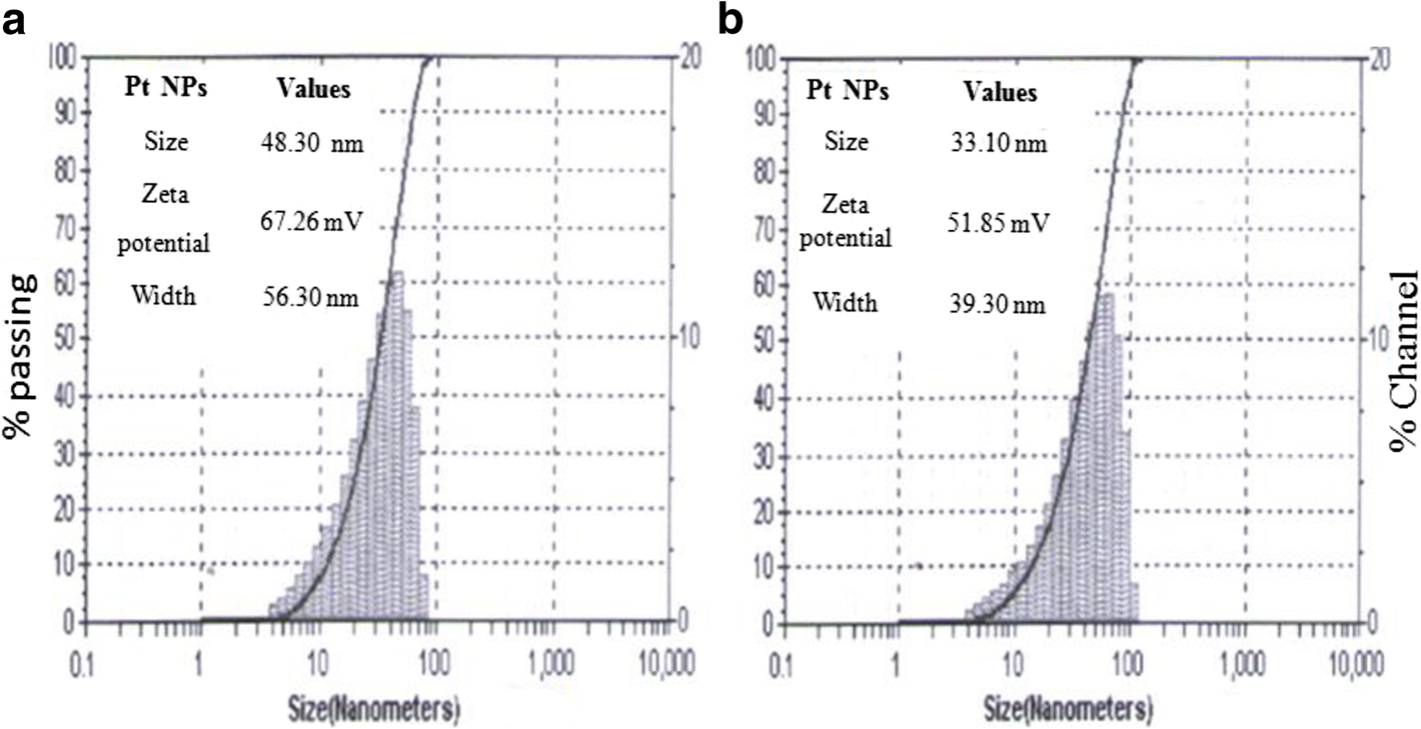
DLS size distribution measurements at 3 and 5 min irradiation

Figure 3 shows the DLS results to demonstrate the stability of Pt NPs capped by PVP molecules. For the investigation of microwave heating effect on size and stability of Pt NPS, the reaction solution was cooled at room temperature and then proceeds for DLS measurements. The results reveal that the Pt colloids consist of 33.10 to 48.30 nm size and 51.85 to 67.26 mV zeta potential, respectively, optimized by continuous flow of MW irradiation at 3 min (Table 1). A larger zeta potential at 3 to 5 min irradiated Pt colloidal suggested a higher charge density of an accumulated n- electron of C=O group of soft PVP molecules on solid surface of Pt NPs [33–35]. Figure 3a, b depicts the size distribution with varying size and zeta potential up to 5 min (Table 1). The result suggested that the size and zeta potential vary with the applied conditions for irradiations which help to fast reduction as Pt NPs and simultaneous capping of PVP [36, 37]. These results support an irradiated effect to form Pt NPs with varying size and higher stability due to the PVP adsorption as donor and Pt surface as accepter.

**Table 1:** DLS based optimized parameters for microwave irradiation

Parameters	Optimized observations	
MW power	180 W	180 W
Temperature	300 °C	300 °C
Reaction time	3 min	5 min
Size	33:10 nm	48:30 nm
Zeta potential	(+) 51:85 mV	(+) 67:26 mV

### HRTEM Analysis of Pt NPs

**Fig. 4:**
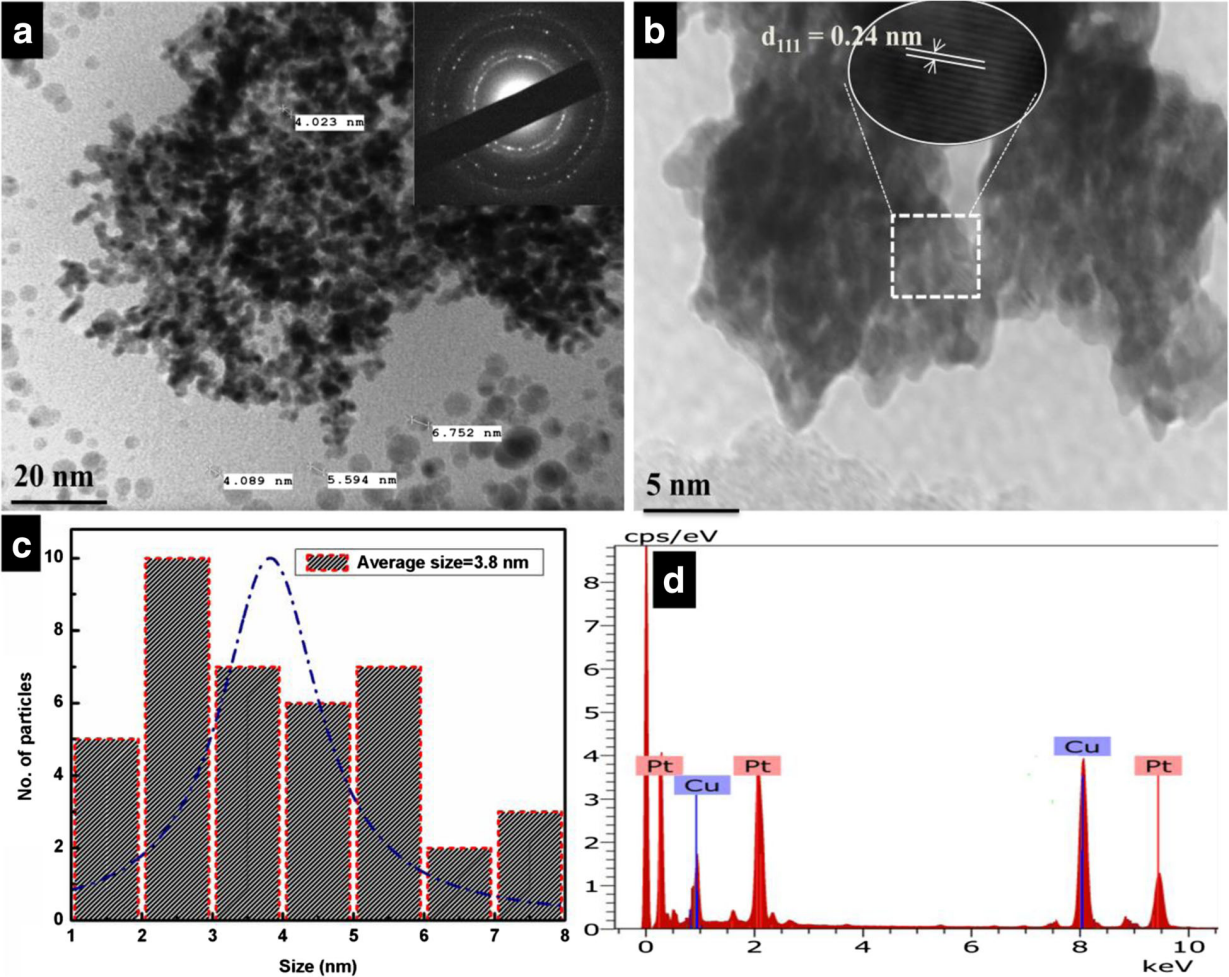
Morphology of Pt NPs with their size, shape, crystallinity, and elemental purity. (**a**) spherical Pt NPs with SAED pattern (**b**) HRTEM image of Pt NPs (**c**) particle size distribution histogram of Pt NPs (**d**) EDS spectrum of Pt NPs

**Fig. 5:**
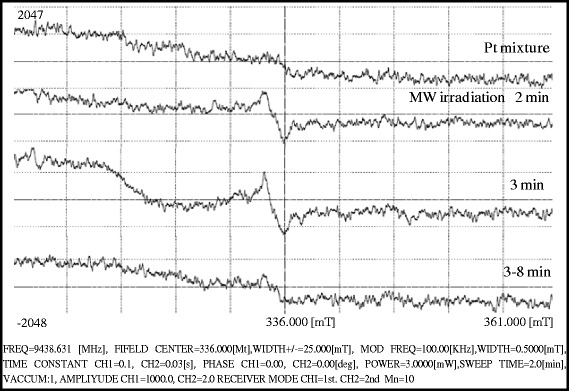
ESR study at 180 W and 300 °C for free radical formation from glycerol

**Fig. 6:**
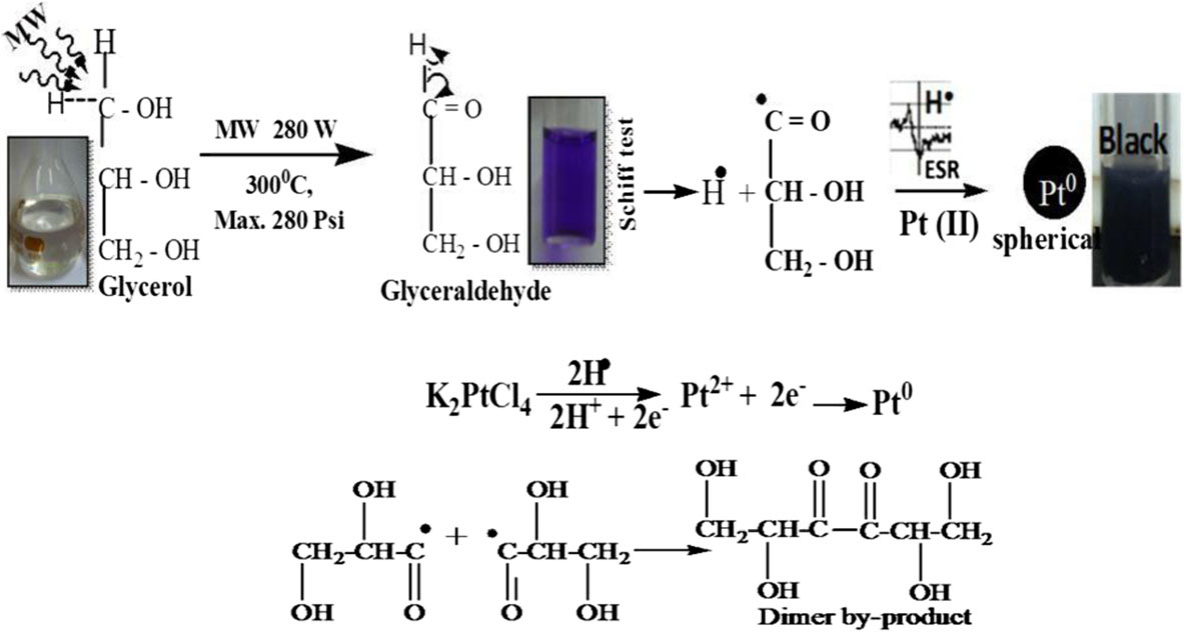
Proposed mechanism for free radical and Pt(0) formation under microwave irradiation

Figure 4 shows HRTEM images and size distribution of platinum colloids synthesized by microwave irradiation. We mounted one drop of Pt dispersion on carbon-coated copper grid and kept it over night at room temperature. HRTEM (JEOL JEM 2100F) at 200 kV was used to investigate morphology of Pt NPs. Spherical-shaped Pt NPs are encaged with the PVP monomers appeared as faceted particles with 2–8-nm diameters (Fig. 4a). The Pt domains showed a polydispersity in distributions of size may be due to the increasing reaction time from 3–5 min, and the average size was 3.8 nm (Fig. 4c). HRTEM studies displayed that these nanoparticles have lattice fringes of 0.23 nm which can be indexed as {111} of FCC Pt (Fig. 4b) [38]. The spherical fine particles are formed under uniformly rapid irradiation with shorter time allowing simultaneous capping of enough PVP monomers on particle exposed surface [39–42]. The crystalline phase was confirmed by SAED, and the pattern (Fig. 4a) showed the higher crystalline particle growth due to uniform irradiation at 300 °C. It is concluded that the higher thermodynamic condition help to generate active free radicals (Fig. 6) which increased fast reaction rate to form Pt(0). Initially, the experiments in microwave were conducted at 100, 200, and 300 °C. The reaction at 300 °C supported breaking of glycerol bonds for radical generation at 280 W under 380 psi pressure in microwave. The 300 °C which is slightly higher than boiling point of glycerol has facilitated bond disruption process. Also, the reaction was conducted up to 400 W of microwave dose and 300 °C (higher thermodynamic), but these conditions aggregated the platinum NPs. So, the 300 °C and 280 W microwave dose was found optimum conditions for platinum NPs preparation. Figure 5d shows elemental compositions identified using HRTEM equipped with an EDS, Pt, and Cu was obtained. However, the Cu shows a peak at 8 KeV due to Cu and the Pt show at 2 and 9.5 KeV, respectively, confirmed the presence of Pt NPs [43].

### ESR Study

Figure 5 shows the ESR spectra of free radical formation during an irradiation. The data were collected at liquid N_2_ environment. The samples were placed in a 4.5-mm diameter quartz Dewar tube. The spectra of continuous-wave ESR (CW-EPR) at 9439.939 MHz, MOD 100.00 KHz, microwave power 3 mW, and the sweep time was 2 min. An effect of applied microwave frequency was studied at 300 °C for 3 min to produce free radicals (Additional file 1: Figure S2). The 280-W frequencies was enough to break C–H bond resulting free radical which exist a high intense ESR peak at 336.000 mT in ESR spectra. The free radical formation from glycerol to glyceraldehyde explained in proposed mechanism (Fig. 6). The ESR peak confirmed free radicals formation from aqueous glycerol solution during the microwave irradiation. Since the EPR is very sensitive towards free radicals, so the EPR for K_2_PtCl_4_ as precursor of Pt^2+^ plus aqueous glycerol was recorded which did not produce any specific signal. But after subjecting aqueous glycerol to microwave at 300 °C which produced free radicals gave specific ESR signal. It has proven that microwave is responsible for free radical formation.

### Microwave-Assisted Optimizing Proposed Mechanism

MW-assisted irradiation method was performed by applying 280 W power at 300 °C temperature inside the microwave chamber with max 280 psi. In this process, firstly, the glycerol breaks down in their possible moieties as radical, ions, or charge species followed by glycerol conversion into glyceraldehyde initially (Additional file 1: Figure S3). The K_2_PtCl_4_ as Pt(II) precursor has large microwave absorption cross section relative to the solvent. It has allowed a quick decomposition of the precursor. As a result, the active moieties (free radicals) of reducing agent started to propagate the mechanism as is shown in Fig. 6. Dissociation of K_2_PtCl_4_ using MW irradiation generates (PtCl_4_)^2−^ and 2 K^+^ ions and highly activated radicals of CH_3_–CH_2_–CHO reduced Pt(II) to zerovalent Pt(0). The glycerol was converted into glyceraldehyde generating free radical investigated with ESR analysis which acts as strong reducing agent for producing Pt neutral atoms. It is seen that glyceraldehyde and hydrogen free radicals were formed on subjecting the mixture to microwave at 300 °C and 280 W. Figure 6 depicts that the hydrogen free radicals reduced the Pt^2+^ to Pt^0^ NPs. The glyceraldehyde free radicals are involved in forming a new product which remains soluble in mixture. The glyceraldehyde (HO–CH_2_–CH–OH–C^•^=O) formed a dimer type glycerol molecules noted as under. It seems that the glyceraldehyde acts as source of H^•^ free radical.

## Electrocatalytic Property as Synthesized Pt NPs for Ethanol Redox Reaction

**Fig. 7:**
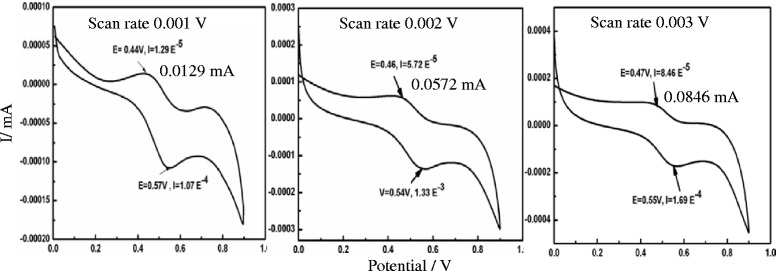
CV study of Pt NPs for ethanol electrocatalytic activity

The cyclic voltammetry measurement was performed using scanning electrochemical microscope (CHI920D). The electrochemical cell was consisted with conventional three electrode system, where the platinum suspension was directly used as working electrode and Ag/AgCl and Pt wire were used as reference and counter electrodes. The CV experiment was performed at room temperature, and the cell was kept on a Faraday cage on top of the optical table to avoid the electronic and acoustic noise. The cyclic voltammetry measurements were performed to investigate the in situ grown Pt NPs under microwave irradiation. Here, we have reported uniformally irradiated Pt NPs colloids directly as electrocatalyst for ethanol redox response. In the CV study, Pt NPs have used as catalyst in range of 0 to 0.9 V in solution of 0.5 M H_2_SO_4_ and 0.5 M ethanol [44]. The CV study was employed at scan rates from 0.001 to 0.003 Vs^−1^ (Fig. 7). The current-potential polarization curve of Pt colloids at 50 mV/s was recorded in range of 0 to 0.9 V. The applied scan rates were 0.001, 0.002, and 0.003 V, and the obtained current densities were 0.0129, 0.0572, and 0.0846 mA at 0.44, 0.46, and 0.55 V, respectively. The CV graph displayed higher current density (0.0846 mA) at 0.55 V which showed the good electrocatalytic performance of used Pt suspension. The CV curves expressed a catalytic response of the in situ grown and directly used Pt NPs by observing high current density. It is concluded that the developed strategy as in situ growth of Pt NPs via free radical formation under microwave not only produced the controlled Pt NPs but also exhibited a good electrocatalytic activity. It could be explained due to (111) plan of spherical Pt NPs having large numbers of active sites on surface [45]. So, we have focused on developing the new route for Pt NPs synthesis and their direct in situ application for electrocatalytic performance. To prepare Pt NPs through ordinary route is a difficult task, so these NPs have been prepared using microwave method. It is also advisable that the Pt salts used as precursors are expensive and highly stable. Thereby, the ordinary methods require multistep activities using their large amount with low yield. However, sol-gel method has been tried for developing stable Pt NPs which is being pursued in the lab and soon could be communicated on a comparative mode. The new route for synthesis of novel metallic nanoparticles showed electrocatalytic response. Broadly our platinum NPs were found comparable with bimetallic or nanocomposite materials [27, 46]. However, the objective of our study was to develop a reduction mechanism of platinum cations via free radicals to synthesize platinum nanoparticles. So, the investigation on comparison scale as are reported in literatures differ our approach.

## Conclusions

Platinum NPs have successfully synthesized under in situ growth mechanism of free radicals. In the chosen method, we had applied 280 W microwave frequencies (300 °C and 280 psi) to produce free radicals from aqueous glycerol. Free radical formation was confirmed at 336.000 mT in ESR spectra. The UV-vis spectra have shown a complete reduction of Pt(II) to Pt(0), and FTIR confirmed simultaneous capping of PVP on Pt surface by C=O and N–C stretching shifts. DLS measurements have shown a good stability of Pt NPs with (+) 57 to (+) 67 mV zeta potential. HRTEM analysis has confirmed spherical nanoparticles under 3.8 nm average size and elemental compositions. The CV studies have shown better electrocatalytic performance for ethanol using as prepared Pt NPs. The single-step method could also be extended for other metallic or multimetallic nanoparticle synthesis and their catalytic applications following this method.

## Additional file

Additional file 1: Figure S1. IR and P-Graph for the microwave reaction system during the reaction. **Table S1.** Optimized conditions for MW irradiation. **Figure S2.** ESR spectra of irradiated aqueous glycerol at 2 to 5 min. **Figure S3.** Schiff test for aldehyde formation under MW heating reaction. **Figure S4.** HRTEM images of Pt NPs. (DOCX 266 mb)
