# Supplementary material for: In Situ Free Radical Growth Mechanism of Platinum Nanoparticles by Microwave Irradiation and Electrocatalytic Properties
Source: Nanoscale Res Lett. 2016 Oct 14;11:458. doi: 10.1186/s11671-016-1653-9 (PMC5065544; doi:10.1186/s11671-016-1653-9)
Supplement: Additional file 1: Figure S1. — IR and P-Graph for the microwave reaction system during the reaction. Table S1. Optimized conditions for MW irradiation. Figure S2. ESR spectra of irradiated aqueous glycerol at 2 to 5 min. Figure S3. Schiff test for aldehyde formation under MW heating reaction. Figure S4. HRTEM images of Pt NPs. (DOCX 266 mb) [file 11671_2016_1653_MOESM1_ESM.docx]

**Supporting information**

**In situ free radical growth mechanism of platinum nanoparticles by microwave irradiation and their electrocatalytic properties**

Gajendra Inwati^1^, Yashvant Rao^1^, Man Singh^1,2*^

^a^Centre for Nanosciences, Central University of Gujarat, Gandhainagar-382030, India.

^b^School of Chemical Sciences, Central University of Gujarat, Gandhainagar-382030, India

Prof. Man Singh

Email: [mansingh50@hotmail.com](mailto:mansingh50@hotmail.com)

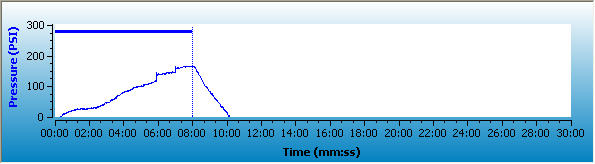


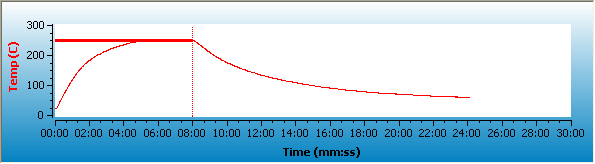


**Figure S1.** IR and P-Graph for the Microwave Reaction System during the reaction

**Table S1** Optimized conditions for MW irradiation

| 1 | Time (min) | MW (Watt) | Temperature (ºC) |
| --- | --- | --- | --- |
| 2 | 2 | 280 | 300 |
| 3 | 3 | 280 | 300 |
| 4 | 5 | 280 | 300 |

**
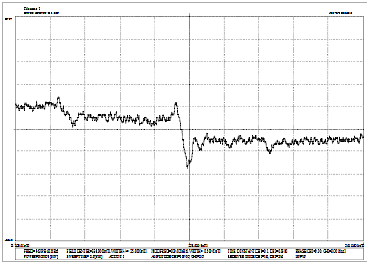
**

**
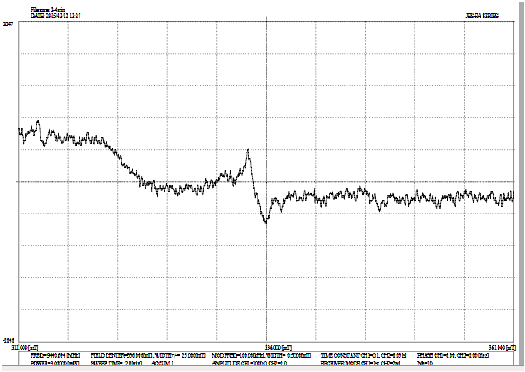
**

**Figure S2:** ESR spectra of irradiated aqueous glycerol at 2 to 5 min


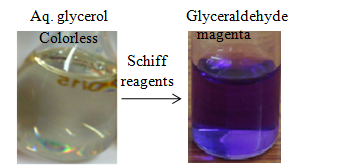


**Figure S3:** Schiff Test for aldehyde formation under MW heating reaction


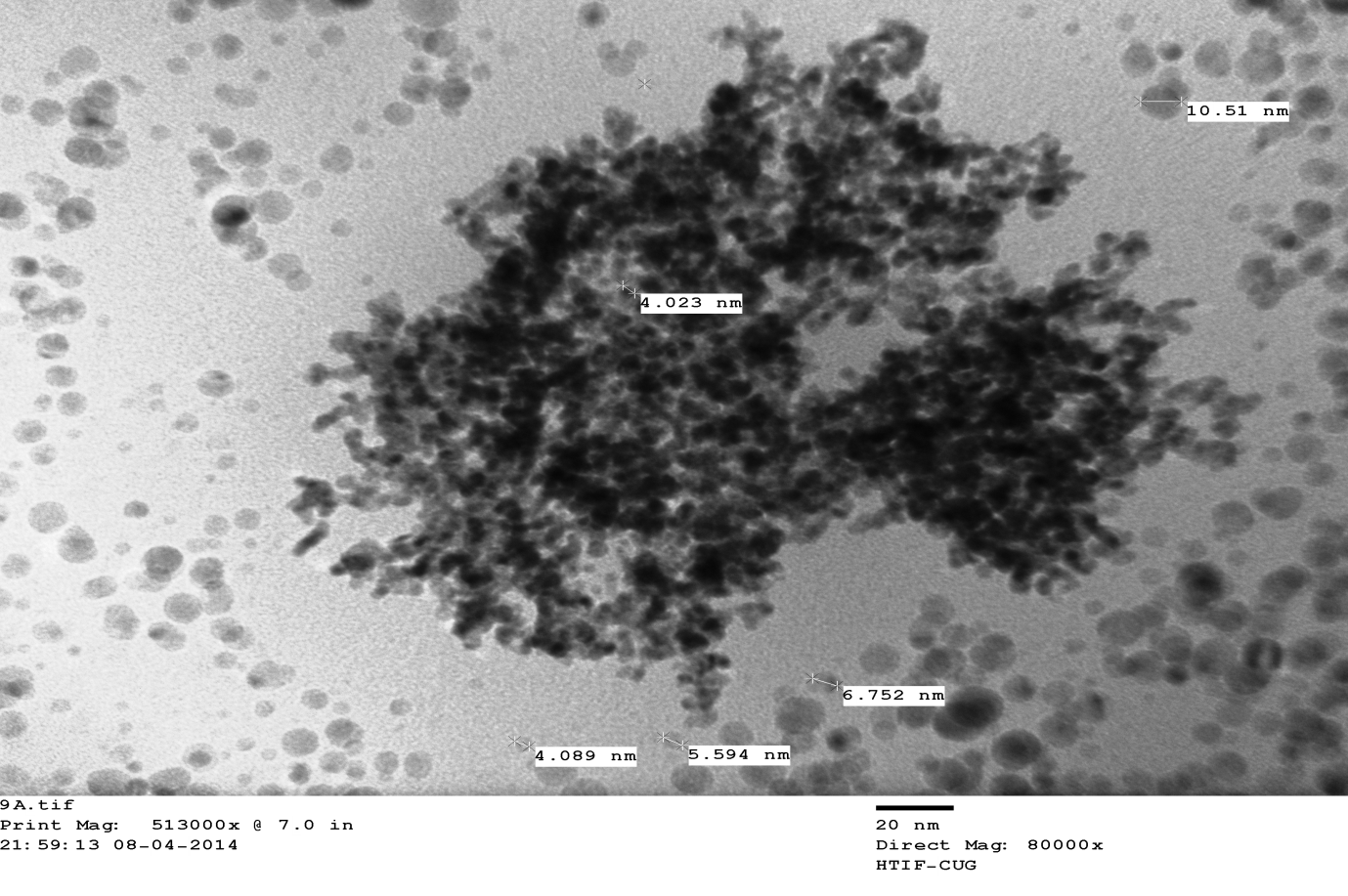





**Figure S4**: HRTEM images of Pt NPs
